# Is upper limb virtual reality training more intensive than conventional training for patients in the subacute phase after stroke? An analysis of treatment intensity and content

**DOI:** 10.1186/s12883-016-0740-y

**Published:** 2016-11-11

**Authors:** Iris Brunner, Jan Sture Skouen, Håkon Hofstad, Jörg Aßmuss, Frank Becker, Hanne Pallesen, Liselot Thijs, Geert Verheyden

**Affiliations:** 1 Department of Global Public Health and Primary Care, University of Bergen, Kalfarveien 31, 5018 Bergen, Norway; 2 Department of Physical Medicine and Rehabilitation, Haukeland University Hospital, Bergen, Norway; 3 Competence Center for Clinical Research, Haukeland University Hospital, Bergen, 5012 Norway; 4 Department of Research, Sunnaas Rehabilitation Hospital, Oslo, Norway; 5 Department of Clinical Medicine, University of Oslo, Oslo, Norway; 6 Hammel Neurorehabilitation Centre and University Research Clinic, Voldbyvej 15, 8450 Hammel, Denmark; 7 Rehabilitation Campus Sint-Ursula, Jessa Hospitals, Herk-de-Stad, 3540 Belgium; 8 Department of Rehabilitation Sciences, KU Leuven, Postbus 1501, Leuven, 3000 Belgium

**Keywords:** Virtual reality, Stroke, Upper limb, Neurorehabilitation, Motor function, Physical therapy, Occupational therapy

## Abstract

**Background:**

Virtual reality (VR) training is thought to improve upper limb (UL) motor function after stroke when utilizing intensive training with many repetitions. The purpose of this study was to compare intensity and content of a VR training intervention to a conventional task-oriented intervention (CT).

**Methods:**

A random sample of 50 video recordings was analyzed of patients with a broad range of UL motor impairments (mean age 61y, 22 women). Patients took part in the VIRTUES trial and were randomized to either VR or CT and stratified according to severity of paresis. A standardized scoring form was used to analyze intensity, i.e. active use of the affected UL expressed in % of total time, total active time and total duration of a training session in minutes, content of training and feedback. Two raters collected data independently. Linear regression models as well as descriptive and graphical methods were used.

**Results:**

Patients in the VR group spent significantly more time actively practicing with an activity rate of 77.6 (8.9) % than patients in the CT 67.3 (13.9) %, (*p =* .003). This difference was attributed to the subgroup of patients with initially severe paresis (*n =* 22). While in VR severely impaired patients spent 80.7 % (4.4 %) of the session time actively; they reached 60.6 (12.1) % in CT. VR and CT also differed in terms of tasks and feedback provided.

**Conclusion:**

Our results indicate that patients with severely impaired UL motor function spent more time actively in VR training, which may influence recovery. The upcoming results of the VIRTUES trial will show whether this is correlated with an increased effect of VR compared to CT.

**Trial registration:**

ClinicalTrials.gov NCT02079103, February 27, 2014.

**Electronic supplementary material:**

The online version of this article (doi:10.1186/s12883-016-0740-y) contains supplementary material, which is available to authorized users.

## Background

Intensity and repetition have been identified as key factors for promoting neural plasticity [1]. It has been stated that Virtual Reality (VR) training using either specially developed systems or off-the-shelf gaming consoles provides the opportunity to achieve many repetitions, salient stimuli and engages the patients in a motivating and intense way [2]. Furthermore, VR is supposed to deliver task-specific training and multi-sensory stimulation [3]. Some evidence has been found that supports the use of VR for improving arm function after stroke [4], although a recent multicenter trial could not corroborate the superiority of a commercial VR gaming system [5]. As VR is becoming progressively more used in neurorehabilitation a more detailed analysis of VR intensity and treatment components is indicated.

Intensity can be expressed as dosage. There is consent that a higher dosage of movement practice can contribute to better outcomes [6, 7]. However, how the term “dosage” should be defined or which factors of dosage are relevant for improved outcome, is unclear. When examining dose–response relationships, Lohse et al. [8] found a positive and significant relationship between amounts of therapy provided and motor function improvement after stroke. However, the authors pointed out the need of a more precise measure of active time and repetitions. In a recent review Lang et al. [9] emphasized the need for a deeper understanding of dose–response relationships. Measuring amount of practice in terms of therapy sessions - scheduled or actually conducted - has been widely used, but does not reflect intensity or active time. It has been demonstrated that patients spend less than two-thirds of their treatment sessions actively and that physiotherapists tend to overestimate the amount of active practice [10, 11]. Many VR systems provide a substantial advantage by the integrated registration of time spent actively practicing and other information on training performance [3, 12].

Like duration of therapy time, also many repetitions of meaningful and challenging exercises are regarded as beneficial for regaining motor skills after stroke [13]. Timmermans et al. [14] identified 15 components to characterize task-oriented training and examined their relation to effect sizes. They found random and distributed practice, clear functional goals and feedback to be associated with larger effect sizes. These components, however, can be found in both VR and conventional training.

Rand et al. [15] used accelerometers to compare the amounts of purposeful movements elicited in a group of patients with stroke using video games and a control group receiving traditional therapy. They found that playing video games resulted in more purposeful repetitions (median 271) than traditional training (median 48). Also cognitive and emotional involvement, considered as key factors for regaining motor skills, may be facilitated by many VR applications due to their playful character [16–18].

The objective of this study was to compare the intensity, here defined as time spent actively using the affected upper limb, and the content of a VR training intervention and a conventional task-oriented intervention. We hypothesized that the intensity of training was higher in the VR group and that patients in VR would achieve more repetitions.

## Methods

### Design

Video recordings of 50 patients with impaired upper limb motor function after stroke in five different rehabilitation sites were obtained, 25 of virtual reality (VR) and 25 of conventional arm training (CT). The patients were a consecutive subsample of those who took part in the VIRTUES trial, where they were randomized to either VR training or conventional training for the upper extremity for four weeks, with details described elsewhere [19]. Patients could be included in the main study within three months post stroke if they had a first ever stroke, or former stroke without motor residuals and did not suffer from major cognitive problems (score of > 20 on MMSE). Inclusion criteria for arm motor function were a score of less than 52 on the Action Research Arm Test (ARAT) and at least 20 degrees of active shoulder extension and abduction. Severe paresis was defined as less than 20 degrees of active wrist and ten degrees of active finger extension, otherwise patients were classified as mild-moderate. Patients were assessed with the Action Research Arm Test (ARAT), Box and Blocks Test (BB) and Functional Independence Measure (FIM) when entering the study. Research staff was asked to videotape one random treatment session during the four-week treatment period with patients from both VR and conventional training and both severity strata.

### Intervention

The intervention lasted for four weeks with four to five training sessions/week of 45 – 60 min duration. Length of an individual session was determined by the patients’ general condition and motivation. Patients in both groups were seated at a table during the training session and received individually tailored exercises for arm and hand movements according to their needs and abilities, based on a pre-defined and standardized treatment program.

Patients randomized to the VR group participated in VR-based training with the YouGrabber system (YouRehab Ltd., Switzerland) which comprises wearable data gloves with sensors and training software with different rehabilitation games. The YouGrabber provides a graded training program of task-related exercises that can be performed unilaterally or bilaterally. Conventional arm training comprised task-related practice for gross movements and dexterity including different grips and selective finger movements, strength training, stretching, and training in daily life activities. Patients in both groups were encouraged to active training. The speed and the number of repetitions were adapted to patients’ actual abilities either automatically by the VR system or the therapist in VR, or the therapist alone in CT.

#### Video analysis

The video recordings were analyzed with regard to active training time, total training time, content of training, number of repetitions, and activity rate, defined as the percentage of time spent actively practicing during a therapy session. Time was registered with a stop watch in the conventional training group. Time was stopped when a task was obviously terminated or when no visible activity was present for more than 2 s. In the VR group active time was automatically generated by the system. A scoring form for video analysis was developed including categories used by Timmermans et al. to describe task-oriented training [14]. Other categories were added, such as mobilization, strength training and unimanual/bimanual training (see Additional file 1). The number of tasks within a training session was registered.

Inspired by procedures applied in former studies by De Wit et al., the following steps were undertaken to assure inter-rater reliability and content validity [20]. Two different raters tried out the scoring form when watching training sessions. They scored independently, compared and discussed their results after each task. As a result, several adjustments of the scoring form were made. During an initial calibration meeting both raters watched training sessions together and compared their assessments immediately. They agreed on procedures to minimize systematic errors. A difference of less than 10% for the measurement of total active time in one session between the raters was regarded as acceptable. After the initial calibration process the raters watched and scored the video recordings independently. The interrater reliability was examined with ICC statistics with excellent agreement (ICC _agreement_ = .98).

#### Data analysis

Descriptive statistics and frequencies were used to describe patient characteristics. To understand the association between treatment and total time (the duration of a training session), active training time (time spent actively during a training session) and activity rate (percentage of time spent actively) we used linear regression models as well as descriptive and graphical methods. We fitted an unadjusted linear regression model with the predictors VR and severe paresis one at a time as well as an adjusted model including both predictors and their interaction. T-tests and Chi-square tests were applied to assess differences in patient characteristics and repetitions. For data analysis SPSS 22 and Matlab 7.10 were used. The significance level was set to 0.05.

## Results

Video recordings of 50 patients (*n =* 25 in VR, *n =* 25 in CT) were analyzed with regard to total training time, active training time, activity rate and content of training. Patient characteristics are presented in Table 1.

**Table 1:** Characteristics of patients in each group

	VR (*n =* 25) Mean (SD)	CT (*n =* 25) Mean (SD)	p
Age, years	59.6 (15.6)	61.6 (12.6)	.62
Days post stroke	50.9 (19.0)	48.6 (24.0)	.71
Days after baseline assessment	22.2 (7.3)	21.0 (9.7)	.77
ARAT (score out of 57)	28.8 (16.1)	23.2 (19.0)	.29
Box and Blocks (blocks per min.)	16.6 (14.4)	12.4 (14.0)	.31
FIM (score out of 126)	91.0 (21.3)	97.7 (21.5)	.28
	n	n	
Sex (male/female)	16/9	12/13	.73
Affected arm (left/right)	15/10	12/13	.57
Paresis (Moderate to mild/severe)	15/10	12/13	.57

*Abbreviations*: *ARAT* Action Research Arm Test, *FIM* Functional Independence Measure, *VR* Virtual reality training

**Fig. 1:**
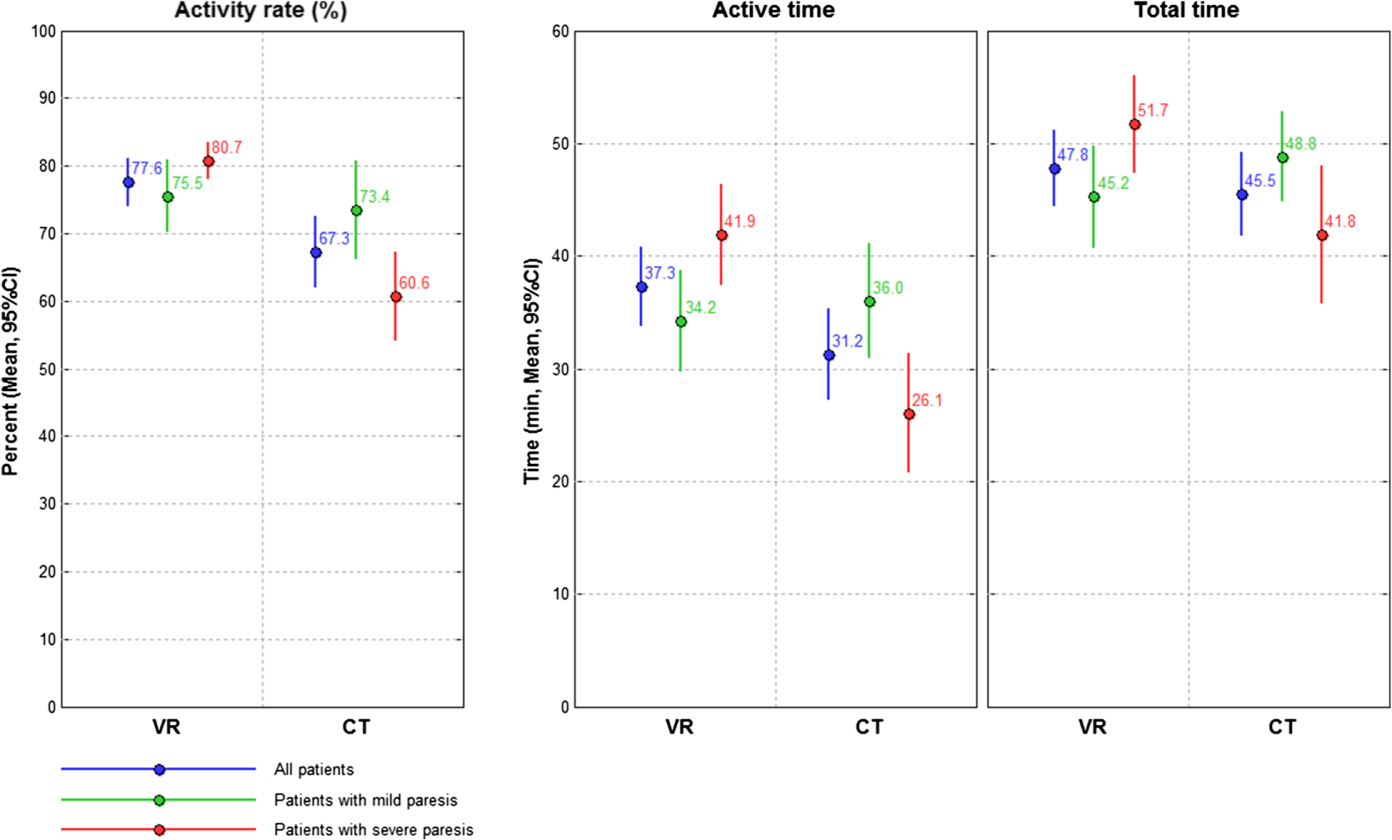
Activity rate, active time and total time for Virtual Training (VR) and conventional training (CT) for all patients (*blue*), and subdivided into patients with mild to moderate (*green*) and severe (*red*) paresis

The groupwise mean time measures are presented in Fig. 1 while the results of the comparison of the treatment groups are presented in Table 2. We observed a significant difference in activity rate of 10.3 % and active time of 6.1 min, while there was no significant difference in total time. This is visible in the univariate analysis, which only shows the differences between VR and CT in the blue graphs in Fig. 1. The multivariate analysis, taking into account the interaction of severity of paresis and kind of intervention received, showed that patients with severe paresis (red) achieved a significantly higher activity rate, while patients with mild paresis (green) did not. The ARAT at study admission had been 7.5 (6.6) for severe and 39.5 (9.6) for patients with mild to moderate paresis. Also active training time and total training time was different for the severely impaired patients only. This effect was significant as shown by the significant interaction in the multivariate analysis.

**Table 2:** Interaction of severity of paresis and Virtual Reality training

Activity rate		Univariate analysis			Multivariate analysis	
Predictors	B	95 % CI	p	B	95 % CI	p
Severe paresis	–4.8	(–12.0, 2.4)	.189	–12.8	(–21.4,–4.1)	.005
VR	10.3	(3.7, 16.9)	.003	2.1	(–6.1, 0.3)	.612
Interaction						
(VR, severe paresis)	-	-	-	15.1	(2.0, 28.2)	.025
Active time	Univariate analysis			Multivariate analysis		
Predictors	B	95 % CI	p	B	95 % CI	p
Severe paresis	–106.5	(–456.6, 243.7)	.544	–595.7	(1022.9,–168.4)	.007
VR	363.5	(30.8, 696.1)	.033	–107.0	(–511.5, 297.4)	.597
Interaction						
(VR, severe paresis)	-	-	-	1057.1	(446.8, 1667.4)	.001
Total time	Univariate analysis			Multivariate analysis		
Predictors	B	95 % CI	p	B	95 % CI	p
Severe paresis	–34.9	(–353.2, 283.4)	.826	–418.5	(–840.2, 3.2)	.052
VR	141.0	(–172.5, 454.6)	.370	–214.7	(–613.9, 184.4)	.285
Interaction						
(VR, severe paresis)	-	-	-	805.6	(203.3, 1407.9)	.010

The content of the training differed in several respects. While the tasks in the VR comprised solely task-related functional movements, tasks in the CT covered a broader range including strength exercises and mobilization. There was no difference between VR and CT with regard to repetitions (*p =* 0.80). However, only discrete functional movements could be counted and not continuous movements which were frequently performed in both groups. Table 3 provides an overview of the content of training in VR and CT. Tasks in VR were shorter, 2–3 min, but frequently repeated during one session and then counted as one task.

**Table 3:** Content of tasks

	VR	CT
Content of tasks	Functional tasks only	Functional tasks (65 %) strength exercises (10 %) mobilization, stretching (23 %) other (2 %)
Repetitions/session mean (SD), discrete movements only	130.05 (217.11)	128.87 (85.10)
Unilateral/bilateral training per session	7.5 tasks unilateral (83 %)	4.9 tasks unilateral (73 %)
	1.5 bilateral (17 %)	1.9 bilateral (27 %)
Use of real life objects	No	In 36 % of the tasks
Feedback	Several modes of feedback, verbal, visual, auditive, tactile	Verbal feedback only
	Knowledge of results and knowledge of performance	Knowledge of results and knowledge of performance

## Discussion

VR training resulted in a higher activity rate per training session. This was especially pronounced for patients with severe paresis who also had longer training sessions and more active time in VR than in CT. For patients with mild to moderate paresis, the difference was present, but less pronounced and not statistically significant. Our results suggest that a higher activity rate was easier to reach in the VR group. This is in accordance with what generally is regarded as a main benefit of VR training [2]. The entertaining and persistent character of the training may facilitate a higher training intensity making it easier to achieve a higher activity rate. VR training normally provides a wide range of stimulating tasks. Interestingly, this seems to be particularly relevant for patients with little motor function. Purposeful, task-related exercises are regarded as most beneficial for arm motor function after stroke [6]. However, it can be very challenging for therapists to find appropriate active exercises for this patient group and to continue training when the patient shows signs of strain. In suitable VR environments patients experience that even small movements can be translated into purposeful actions. A sense of achievement, to be able to do something with an otherwise useless hand can be very motivating for patients with severe paresis. Furthermore, the multimodal feedback provided by the VR system may facilitate cerebral reorganization in a phase where highest plasticity can be expected [21].

In general, our patients were active during 72 % their training sessions, which is more than identified in a previous review article where only 60 % active time was found [10]. Although the exact dose–response of rehabilitative treatment approaches is still unclear, the results of several reviews indicate a beneficial effect of augmented training time on functional outcomes and activities of daily living [7–9]. It is recommended to find ways to increase therapy time after stroke and VR training seems to be an appropriate treatment alternative which also can increase adherence to exercises. Our intervention took place in a clinical setting under the supervision of a therapist, which facilitated adherence. However, in a home setting without supervision, it may be difficult to achieve recommend levels as demonstrated in a study by Standen et a. [22] Active time in therapy expressed as activity rate was the main focus of the current study. Measuring activity rate seems to be the most objective and tangible aspect of motor training. Comparing VR to dose-matched interventions in terms of scheduled therapy sessions only may not reflect the real extent of activity. [23] There was no difference for total time and active time between VR and CT when looking at all patients, which was expected since the training in both groups was supposed to be matched for these parameters per protocol. However, for the more impaired patients also longer total time was revealed, not only a higher activity rate and longer active time. Longer sessions in VR may indicate that it was easier to perpetuate training for a longer time.

While our main focus was on training aspects related to time, we also intended to explore other features of the training provided in both groups. Counting repetitions for discrete functional movements was of very limited value in this study. The number of repetitions did not reflect activity rate or intensity, since both the CT and the VR training comprised continuous tasks, such as steering a plane (VR) or cleaning a table or writing (CT). VR comprised probably a larger part of continuous tasks that could not be counted as repetitions. Severely impaired patients with no grasp function frequently had no repetitions or no grasps registered, although they had been active through large parts of the training session. Training in VR consisted of exclusively functional tasks, while training in CT also comprised other tasks, mainly mobilization. However, repetitions of discrete functional movements registered were substantially, roughly 3 times, higher in both VR and CT than those registered by Kimberley et al. [24], who found only a mean 40.64 repetitions per session. An also relatively low number of only 39 active and 12 purposeful movements per session were counted by Lang et al. [25]. Another study by Lang et al. revealed that functional UL movements occurred in 51 % of training sessions [26]. Comparison with these earlier results indicates that our patients both achieved a very high activity rate and relatively many repetitions. Still, the activity rate and number of repetitions does not come close to what is regarded as facilitating neural plasticity based on animal studies [21, 27]. Activity rate seems to be the closest we can get to gauge intensity. However, it has been demonstrated that motor learning and cortical reorganization is not based on stereotype repetitions and the simple use of the hand [28]. Plastic changes only occur when skilled tasks are practiced, and may not automatically generalize to untrained tasks [29, 30]. Motivation and engagement can facilitate general motor learning and retention [31]. It has been claimed that these requirements can be met by enriched virtual environments rather than by conventional therapy [2].

We also registered the kind of feedback delivered. The benefit of explicit feedback is still unclear. In some studies a beneficial effect of knowledge of performance on motor learning was found, while other studies suggest that explicit information interferes with implicit learning [32, 33]. In VR feedback was provided in a multi-modal way, since the patients could see scores reached, heard applause, felt vibrations on the hand. Also the training report that describes progress over a series of sessions can be regarded as feedback. Verbal feedback from the therapist comprising knowledge of performance and knowledge of results was provided in both groups. However, knowledge of performance with the intention to control for excessive compensatory trunk movement was provided in CT only. This may have allowed for more compensatory strategies in VR [32]. However, knowledge of performance can also be provided in VR by therapists or included as a part of the virtual environment as in the study by Subramanian et al. [34].

The fact that a session was video-recorded may have influenced both the patient and the therapist and could have resulted in altered behavior [35] and most likely in a way that effective training time was increased and small talk and extensive breaks discouraged. This may have affected CT more, since in VR the course of the sessions including breaks usually was pre-programmed. Since our study was cross-sectional we cannot tell if more was better in this case. Instead we have to wait for the results of the main study. The motor abilities of our patients were not assessed at the day of the video-recording. Therefore we cannot tell how much the initially severe motor impairment had improved that special day. We also have to acknowledge that the initial categorization based on active finger and wrist extension can be debated. However, we can tell from the video-recordings that patients with initially severe arm motor impairment still suffered from pronounced paresis at the day of recording. Although the video recordings were taken at a random day, we cannot rule out to have picked a session that was not quite representative for all sessions of this respective patient. Large deviations would have been reported by the therapist.

## Conclusion

A markedly higher activity rate for patients with severe paresis in the VR group suggests that VR training facilitates more active training time. Also a longer active time and total time was registered for patients with severe impairments, indicating that it may be easier to adhere to the training. Only functional tasks were observed during training period in the VR group compared to only 65 % of the time in the CT group. Several modes of feedback were registered in the VR group. It remains to find out if these differences in quality of training between the programs will cause any difference in arm function.

## Additional files

Additional file 1: The scoring form. (PDF 145 kb)
Additional file 2: Data file. (XLS 25 kb)

## Abbreviations

ARAT: Action Research Arm Test
CT: Conventonal training
FIM: Functional Independence Measure
VR: Virtual Reality training
