# Supplementary material for: Is upper limb virtual reality training more intensive than conventional training for patients in the subacute phase after stroke? An analysis of treatment intensity and content
Source: BMC Neurol. 2016 Nov 11;16:219. doi: 10.1186/s12883-016-0740-y (PMC5106796; doi:10.1186/s12883-016-0740-y)
Supplement: Additional file 1: — The scoring form. (PDF 145 kb) [file 12883_2016_740_MOESM1_ESM.pdf]

Appendix 1. Scoring form

Pasient-ID:……   Total time:…………   Effective time total:…………   Total tasks:………

**Task/game number** **Task description**		1	2	3	4	5	6	7	8	9	10
**Time** **Number of reps**											
**Functional tasks** (register time used) (Choose either functional tasks, single plane movement/single joint movement of strength-exercise)	Reach within one armlength in combination with fine motor skills (grasp, pincergrip)										
	Reach more than one armlength away in combination with fine motor skills.										
**Mobilization/warm up/single joint movement** (register time used) (repetitive movements without a goal)	Transversal										
	Frontal										
	Sagittal										
**Strength-exercise** (register time used)											
**Exercise progression**	Yes										
	No										

**Unimanual** (Is mainly the affected arm / hand used to perform the task?) **Bimanual** (Are both hands used, either simultaneously or alternatingly?)	Unimanual										
	Bimanual										
**Real-life object manipulation**	Yes										
	No										
**Which body parts are moved? (predominantly)** (max 3 each side)	Arm affected Lower arm affected (sup./pron)										
	Hand affected Single fingers aff.										
	Arm unaffected										
	Lower arm unaff. (sup./pron)										
	Hand unaff.										
	Single fingers unaff										
**Feedback**	Verbal										
	Visual										
	Auditiv										
	Tactile										
**Task/game number**		1	2	3	4	5	6	7	8	9	10
